# Prevalence, Risk Factors, and Complications of Diabetes in the Kilimanjaro Region: A Population-Based Study from Tanzania

**DOI:** 10.1371/journal.pone.0164428

**Published:** 2016-10-06

**Authors:** John W. Stanifer, Charles R. Cleland, Gerald Jamberi Makuka, Joseph R. Egger, Venance Maro, Honest Maro, Francis Karia, Uptal D. Patel, Matthew J. Burton, Heiko Philippin

**Affiliations:** 1 Division of Nephrology, Department of Medicine, Duke University, Durham, North Carolina, United States of America; 2 Duke Global Health Institute, Duke University, Durham, North Carolina, United States of America; 3 Duke Clinical Research Institute, Duke University, Durham, North Carolina, United States of America; 4 Eye Department, Kilimanjaro Christian Medical Centre, Moshi, Tanzania; 5 Kilimanjaro Christian Medical University College, Moshi, Tanzania; 6 International Centre for Eye Health, London School of Hygiene & Tropical Medicine, London, United Kingdom; 7 Moorfields Eye Hospital, London, United Kingdom; Florida International University Herbert Wertheim College of Medicine, UNITED STATES

## Abstract

**Background:**

In sub-Saharan Africa, diabetes is a growing burden, yet little is known about its prevalence, risk factors, and complications. To address these gaps and help inform public health efforts aimed at prevention and treatment, we conducted a community-based study assessing diabetes epidemiology.

**Methods and Findings:**

We conducted a stratified, cluster-designed, serial cross-sectional household study from 2014–2015 in the Kilimanjaro Region, Tanzania. We used a three-stage cluster probability sampling method to randomly select individuals. To estimate prevalence, we screened individuals for glucose impairment, including diabetes, using hemoglobin A1C. We also screened for hypertension and obesity, and to assess for potential complications, individuals with diabetes were assessed for retinopathy, neuropathy, and nephropathy. We enrolled 481 adults from 346 urban and rural households. The prevalence of glucose impairment was 21.7% (95% CI 15.2–29.8), which included diabetes (5.7%; 95% CI 3.37–9.47) and glucose impairment with increased risk for diabetes (16.0%; 95% CI 10.2–24.0). Overweight or obesity status had an independent prevalence risk ratio for glucose impairment (2.16; 95% CI 1.39–3.36). Diabetes awareness was low (35.6%), and few individuals with diabetes were receiving biomedical treatment (33.3%). Diabetes-associated complications were common (50.2%; 95% CI 33.7–66.7), including renal (12.0%; 95% CI 4.7–27.3), ophthalmic (49.6%; 95% CI 28.6–70.7), and neurological (28.8%; 95% CI 8.0–65.1) abnormalities.

**Conclusions:**

In a northern region of Tanzania, diabetes is an under-recognized health condition, despite the fact that many people either have diabetes or are at increased risk for developing diabetes. Most individuals were undiagnosed or untreated, and the prevalence of diabetes-associated complications was high. Public health efforts in this region will need to focus on reducing modifiable risk factors, which appear to include obesity, as well as early detection that includes increasing awareness. These findings highlight a growing urgency of diabetes prevention in this region as well as the need for treatment, including management of complications.

## Introduction

Non-communicable diseases (NCDs) are a growing global epidemic that disproportionately affect economic and health outcomes in low- and middle-income countries (LMICs) [1, 2]. In order to address this disparity, it will be critical to reduce the burden of NCDs in sub-Saharan Africa (SSA) where they are now a leading cause of death among adults [1–3].

In SSA, where more than 900 million people live, expanding population growth is juxtaposed with rapid urbanization [4, 5]. In the context of NCDs such as diabetes, the accompanying lifestyle and dietary changes that parallel this demographic transition mean that millions are and will be at risk [5–7]. As such, current estimates portend that the prevalence of diabetes and glucose impairment in SSA could nearly double by the year 2045, which means that more than 40 million people would be living with diabetes in SSA. Moreover, nearly three-fourths of diabetes-related deaths occur in economically-productive persons under the age of 60 years [6].

One of the most significant barriers in addressing diabetes in SSA is the limited understanding of its epidemiology including community prevalence, modifiable risk factors such as diet and obesity, and complications arising from under-recognition or under-treatment [3, 6, 8–10]. At this time, the prevalence of undiagnosed diabetes in SSA is estimated to be higher than in any other region of the world with as many as two-thirds of cases being unrecognized [6, 10]. Furthermore, despite the high morbidity and mortality of diabetes, the micro-vascular complications resulting from diabetes in SSA are not well-studied due to the lack of comparable epidemiological data [6, 10–12].

To address these gaps, the Comprehensive Kidney Disease Assessment for Risk Factors, epidemiology, Knowledge, and Attitudes (CKD-AFRiKA) study was conducted in the Kilimanjaro Region of northern Tanzania between 2014 and 2015 [13, 14]. Its overall objective was to understand the population-based epidemiology, awareness, and practices associated with NCDs, including diabetes. As part of the CKD-AFRiKA Study, we conducted a cross-sectional, community-based study of diabetes to assess its prevalence, modifiable risk factors including obesity, and end-organ micro-vascular complications, including nephropathy, retinopathy, and neuropathy.

## Methods

### Ethics Statement

The study protocol was approved by Duke University Institutional Review Board (#Pro00040784), the Kilimanjaro Christian Medical College (KCMC) Ethics Committee (EC#502), and the National Institute for Medical Research (NIMR) in Tanzania. Written informed consent (by signature or thumbprint) was obtained from all participants, and all participants with abnormal findings received counselling, educational pamphlets, and reimbursement with referral for follow-up.

### Study Setting

We conducted a stratified, cluster-designed, serial cross-sectional household survey between January and June 2014 and between March and June 2015 in the Kilimanjaro Region of Tanzania. The adult regional population is more than 900,000 people of whom 35% live in urban settings. Most adults have only a primary school level of education (77%). The regional HIV prevalence is 3–5%. The largest ethnic group is the Chagga tribe followed by the Pare, Sambaa, and Maasai tribes. Swahili is the major language [15, 16].

**Fig 1:**
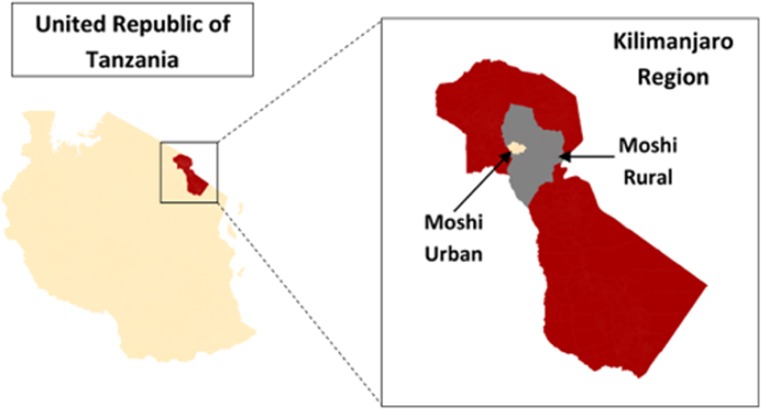
Study Setting. Map showing the sampling area of Moshi Urban and Moshi Rural in the Kilimanjaro Region of Northern Tanzania. *Source*: *Stanifer JW*, *Maro V*, *Egger J*, *Karia F*, *Thielman N*, *Turner EL*, *et al*. *The Epidemiology of Chronic Kidney Disease in Northern Tanzania*: *A Population-Based Survey*. *PLOS ONE*. *2015;10(4)*:*e0124506*. *Open access distribution under the Creative Commons Attribution*, *Non-Commercial*, *No Derivatives 4*.*0 International License*

The region comprises seven districts, and our study was conducted in two of these districts, Moshi Urban and Moshi Rural, which served as strata for our sampling scheme (**Fig 1**). The districts are divided into administrative wards, and each ward is further sub-divided into neighbourhoods, which are the smallest governmental administrative unit in Tanzania. Neighbourhoods range in size from 500 to 5000 people [16].

### Sampling Methods

We used a three-stage cluster sampling method, stratified by urban and rural districts. We used a random-number generator to select twenty nine neighbourhoods within the Moshi Urban and Moshi Rural districts. We based the random neighborhood sampling on probability proportional to size according to the 2012 national census [16]. From the twenty-nine neighborhoods, we then randomly selected the starting point for each sampling area (37 in total) using geographic coordinates randomly generated by Arc Global Information Systems (ArcGIS), v10.2.2 (Environmental Systems Research Institute, Redlands, CA). From the randomly-selected geographic starting point, we then chose households based on a coin-flip and die-rolling technique (**S1 Appendix**). All non-pregnant adults (age ≥ 18 years old) living in the selected households were recruited. To be eligible each adult had to be a permanent resident (living ≥ nine months each year in Tanzania) or a citizen of Tanzania. To ensure that the protocol was strictly adhered to, the principal investigator (JWS) regularly accompanied the surveyors and frequently conducted anonymous shadowing.

We targeted between 15 and 25 participants per sampling area based on the requirements of the CKD AFRiKA study, which was designed to estimate the community prevalence of chronic kidney disease (CKD), diabetes, and hypertension with a precision of 5% when accounting for the cluster-design effect, assuming a disease prevalence up to 20% and an intra-cluster correlation (ICC) coefficient of 0.05. To reduce non-response rates, we attempted a minimum of two additional visits during off-hours (evenings and weekends) and multiple phone calls using mobile phone numbers. When available, we collected demographic data for the non-responders including age, gender, and occupation.

### Data Collection

Between January and June 2014, each participant completed a demographic and medical history survey, was measured for anthropomorphic data, and was tested for CKD, diabetes mellitus, and hypertension. Disease awareness was defined as giving a self-reported disease history and subsequently testing positive during the screening process.

Anthropomorphic data included height, weight, and body mass index (BMI). Normal weight was defined as a BMI of 20 to 24.9 kg/m^2^. Overweight was defined as a BMI ≥25 kg/m^2^ and obesity was defined as a BMI ≥30 kg/m^2^.

Hemoglobin (Hb) A1c was measured from a fingerstick whole blood sample using the Bayer A1c Now+ point-of-care device (Bayer Healthcare LLC; Sunnyvale, CA). Quality control measures were performed weekly according to the manufacturer’s recommendation. Glucose impairment was defined as an HbA1C >6.0% in the presence or absence of ongoing treatment with anti-hyperglycemic medications. Diabetes mellitus was defined as an HbA1c level ≥7.0% or current known use of anti-hyperglycemic medications for the purpose of treating diabetes. Participants with an HbA1C between 6.0% and 6.9% in the absence of treatment with anti-hyperglycemic medications were considered to have glucose impairment with an increased risk for developing diabetes [17].

CKD was defined according to the Kidney Disease Improving Global Outcomes Working Group guidelines [18]. To estimate glomerular filtration rate (eGFR), we used the Modification of Diet in Renal Disease (MDRD) equation without the race factor. A urine albumin greater than 30 mg/dL in the absence of gross haematuria or an ongoing urinary tract infection as confirmed by urinalysis (Siemens Multistix 10G Urinalysis test strips) was considered positive. Urine albumin was detected with a Siemens MicroAlbustix (Siemens Healthcare Diagnostics, Inc.; Tarrytown, NY) from a mid-stream urine sample. All positive urine albumin results were confirmed with a repeat measurement from a first-morning void, mid-stream urine sample more than 48 hours from the initial measurement. Quality control measures were performed weekly on each open and new bottle of urine dipsticks according to the manufacturer’s recommendation. The serum creatinine was measured using the Cobas Integra 400 Plus (Roche Diagnostics; Basel Switzerland) at the Kilimanjaro Christian Research Institute Biotechnology Laboratory. The laboratory is known in the region for its high quality results, and it participates fully in international external quality assurance programs including the College of American Pathologists and the United Kingdom External Quality Assessment Service. All laboratory investigations were conducted according to Good Clinical Laboratory Practice standards.

We measured blood pressure using the automated Omron HEM-712 sphygmomanometer (Omron Healthcare, Inc.; Bannockburn, IL), which has an adjustable cuff size. The machine was calibrated monthly during data collection. All participants were seated in an erect position with feet flat on the floor for a minimum of five minutes before measurements. Hypertension was defined as a single blood pressure measurement of greater than 160/100 mmHg, a two-time average measurement of greater than 140/90 mmHg, or current known use of anti-hypertensive medications.

Between March and June 2015, participants with diabetes mellitus were also screened for retinopathy and peripheral neuropathy. A trained retinal photographer captured fundus images of both eyes using a Topcon retinal camera (TRC NW8S; Topcon Corporation, Tokyo, Japan) following pupil dilation with topical tropicamide 1%. Each digital photograph was graded for retinopathy and maculopathy according to the minimum dataset outlined by the English and Wales National Screening Committee [19]. Two consultant ophthalmologists independently graded each digital photograph and any disagreements were resolved by joint consensus. Retinopathy was graded as absent (R0), background (R1), pre-proliferative (R2), or proliferative (R3). Maculopathy was graded as absent (M0) or present (M1). For each participant, retinopathy and maculopathy were each scored according to the more severely diseased eye.

A single physician assessed for distal, lower-extremity peripheral neuropathy using a scored combination of vibration perception, pinprick sensation, and Achilles ankle reflexes (**S1 Appendix**) [20]. Based on these assessments, a total score (0–8) was reported for each participant. We considered peripheral neuropathy present if the total score was ≥4 [20].

### Data Analysis and Data Management

Data were analyzed using STATAv.13 (STATA Corp., College Station, TX). Continuous variables were summarized by the median and inter-quartile range (IQR). Categorical variables were summarized using counts and percentages. We used a Chi-squared goodness-of-fit test or Fisher’s exact test to compare differences between groups. To account for the design effect on variance due to cluster sampling, we first estimated the level of clustering which occurred at each of the three sampling levels (neighborhood, sampling area, and household) by fitting a mixed effects model with separate random intercepts for neighborhood, sampling area, and household for diabetes and glucose impairment separately. In these models, after accounting for the neighborhood clustering, very little variance in the clustering (<15%) remained at the sampling area level and household level, which indicated to us that most of the variation in these outcomes was explained at the individual- and neighborhood-levels. As such, we estimated the ICC for the neighborhood clusters only. ICC coefficients for the binary outcomes, diabetes and glucose impairment with increased risk for diabetes, using a one-way random effects analysis of variance (ANOVA) estimator [21, 22].

To account for the design effect at each sampling level, all prevalence and model estimates were performed within the ‘svy’ command in STATA, which allowed us to account for variance due to the complex three-level hierarchal survey design. Crude and adjusted prevalence risk ratios (PRR) were estimated using generalized linear models with a log link. All p-values are two-sided at a 0.05 significance level.

To address potential non-response bias in the community-based prevalence estimates, we explored difference in age, gender, occupation, and education between the study population and the regional population, and we sample-adjusted all the prevalence estimates using age- and gender-frequency weights based on the 2012 urban and rural district-level census data [16]. To address potential non-response bias in the estimation of prevalence for diabetes-associated complications, we explored differences in age, gender, HbA1c values, BMI, and serum creatinine between participants who did and did not present for follow-up assessment for retinopathy and neuropathy.

A secondary aim of the analysis was to explore the association between glucose impairment and overweight or obesity status. Step-wise model specification was carried out by using substantive knowledge of potential confounding factors potentially associated with risk of glucose impairment and being overweight or propoobese, such as age, gender, and ethnicity. Candidate models were then compared by calculation of the mean squared error of the effect. We did not include lifestyle or environmental factors, such as urbanicity, occupation, or education in our models due to *a priori* assumptions about their potential upstream causal association with overweight or obesity status.

All data were collected on paper and then electronically entered into and managed using REDCap electronic data capture tools hosted at Duke University. REDCap is a secure, web-based application designed to support data capture for research studies [23]. All data were verified after electronic data entry by an independent reviewer to ensure accuracy.

## Results

We enrolled 481 adults from 346 households (**Table 1**). The median age was 45.0 years (IQR 35–59). The majority of participants lived in an urban district (n = 370; 77.0%), were women (n = 358; 74.4%), ethnically Chagga (n = 288; 59.9%), and had a primary school level of education (n = 349; 72.6%). Participants were most frequently occupied as farmers or daily wage-earners (n = 199; 41.4%). Many participants reported ongoing use of alcohol (n = 198; 41.2%).

**Table 1:** Participant characteristics by Diabetes and Diabetes Risk Status; CKD-AFRiKA study population, 2015;N = 481.

Variable	Participants				
	Overall(n = 481)	No Glucose Impairment(n = 352)	Glucose Impairment(n = 129)		p-value±
			Increased risk for diabetes(n = 84)	Diabetes(n = 45)	
**Gender**					0.81
Male	123 (25.6%)	89 (25.3%)	19 (22.6%)	15 (33.3%)	
Female	358 (74.4%)	263 (74.7%)	65 (77.4%)	30 (66.7%)	
**Age**					<0.01
18–39 years old	172 (35.8%)	143 (40.6%)	24 (28.6%)	5 (11.1%)	
40–59 years old	191 (39.7%)	135 (38.4%)	37 (44.1%)	19 (42.2%)	
60+ years old	118 (24.5%)	74 (21.0%)	23 (27.4%)	21 (46.7%)	
**Setting (District)**					0.50
Rural	111 (23.1%)	84 (23.9%)	21 (25.0%)	6 (13.3%)	
Urban	370 (76.9%)	268 (76.1%)	63 (75.0%)	39 (86.7%)	
**Ethnicity**					0.36
Chagga	288 (59.9%)	217 (61.7%)	45 (53.6%)	26 (57.8%)	
Pare	66 (13.7%)	43 (12.2%)	19 (22.6%)	4 (8.9%)	
Sambaa	27 (5.6%)	22 (6.3%)	3 (3.6%)	2 (4.4%)	
Other*	100 (20.8%)	70 (19.9%)	17 (20.2%)	13 (28.9%)	
**Education**					0.75
None	31 (6.4%)	21 (6.0%)	4 (4.8%)	6 (13.3%)	
Primary	349 (72.6%)	260 (73.9%)	63 (75.0%)	26 (57.8%)	
Secondary	74 (15.4%)	52 (14.8%)	12 (14.3%)	10 (22.2%)	
Post-Secondary	27 (5.6%)	19 (5.3%)	5 (6.0%)	3 (6.7%)	
Occupation					<0.01
Unemployed	74 (15.4%)	52 (14.8%)	15 (17.9%)	7 (15.6%)	
Farmer/Wage Earner	199 (41.4%)	157 (44.6%)	32 (38.1%)	10 (22.2%)	
Small Business/Vendors	158 (32.8%)	121 (34.4%)	25 (29.8%)	12 (26.7%)	
Professional^†^	50 (10.4%)	22 (6.3%)	12 (14.3%)	16 (35.7%)	
**Ongoing Alcohol Use**	198 (41.2%)	157 (44.6%)	33 (39.3%)	8 (17.8%)	0.01
**Ongoing Tobacco Use**	50 (10.4%)	42 (11.9%)	7 (8.3%)	1 (2.2%)	0.07
**Co-Morbid Health Conditions**					
Hypertension	149 (31.0%)	96 (27.3%)	29 (34.5%)	24 (53.3%)	<0.01
BMI (median)	26.2	25.1	29.1	27.8	<0.01
*Underweight*	59 (12.3%)	51 (45.5%)	4 (4.7%)	4 (8.9%)	
*Normal weight*	143 (29.7%)	122 (34.6%)	13 (15.5%)	8 (17.8%)	
*Overweight/Obese*	279 (58.0%)	179 (50.9%)	67 (79.8%)	33 (73.3%)	
HbA1C (median)	5.7	5.5	6.3	9.8	<0.01

±p-value comparing differences by presence or absence of glucose impairment

*Other ethnicities includes Maasai, Luguru, Kilindi, Kurya, Mziguwa, Mnyisanzu, Rangi, Jita, Nyambo, and Kaguru.

† Professional included any salaried position (e.g. nurse, teacher, government employee, etc.) and retired persons.

The household non-response rate was 15.0% and the individual non-response rate was 20.6%. Compared to the regional population [16], men (p<0.001) and young adults 18–39 years old (p = 0.001) were more likely to be non-responders in our study, and the proportion of participants with a secondary or post-secondary education (22.4%) was higher than the regional average (14.6%) (p = 0.02). We observed no significant differences in occupation between the responders and non-responders (p = 0.64). Among participants with diabetes, 35 (77.8%) were screened for retinopathy and 33 (73.3%) were screened for neuropathy. Reasons for non-response in this group were refusal, lost to contact, and illness. There were no significant differences in age, gender, HbA1c, BMI, or serum creatinine (p>0.05 for all) between those with diabetes who responded and those who did not respond for follow-up assessment.

The overall prevalence of glucose impairment was 21.7% (95% CI 15.2–29.8), which included diabetes and glucose impairment with increased risk for diabetes. The community prevalence of diabetes was 5.7% (95% CI 3.37–9.47), and the prevalence of glucose impairment with increased risk for diabetes was 16.0% (95% CI 10.2–24.0). The neighborhood-level ICC coefficient for diabetes was <0.001, and the neighborhood-level ICC coefficient for glucose impairment with increased risk for diabetes was 0.031.

Individuals with glucose impairment were older, more commonly occupied as professionals, had hypertension, or had an overweight or obese BMI (p<0.01 for all), and they were less likely to be consuming alcohol (p = 0.01). Among the 45 individuals with diabetes, 30 (66.7%) were female, most had a primary school level of education or less (n = 26; 56.8%) and lived in an urban setting (n = 39; 86.7%), and many were occupied as professionals (n = 16; 35.7%) (**Table 1**). The median age was 57 years (IQR 48–67). The median HbA1C of participants with diabetes was 9.8% (IQR 7.5–11). Over half of the individuals with diabetes also had hypertension (n = 24; 53.3%), and almost three-quarters (n = 33; 73.3%) were overweight or obese. The median BMI of individuals with diabetes was 27.8 (IQR 24.7–30.1); however, several individuals (n = 12; 26.7%) had a normal weight BMI (n = 8; 17.8%) or underweight BMI (n = 4; 8.9%). Among the 84 individuals who had glucose impairment with an increased risk for diabetes, 65 (77.4%) were female, and the median age was 51.0 years (IQR 37.5–60). The median HbA1C was 6.3% (IQR 6.2–6.5), and the median BMI was 29.1 (IQR 25.7–32.0).

**Fig 2:**
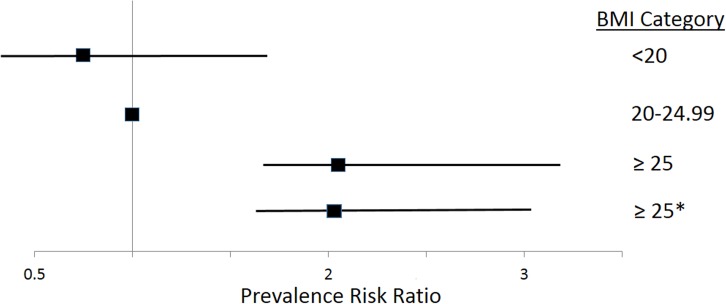
Forest Plot. Unadjusted and adjusted prevalence risk ratios, with corresponding 95% CI, for glucose impairment by body mass index (BMI) category. *Adjusted for age, gender, and ethnicity.

Overweight or obesity status was an independent risk factor for glucose impairment (**Fig 2**). In univariable regression modelling, compared with normal weight individuals, there was an increased prevalence risk (PRR 2.16; 95% CI 1.39–3.36) of glucose impairment associated with being overweight or obese (BMI ≥25). Adjustment for potential confounders in multi-variable modelling, including age, gender, ethnicity, and ongoing biomedicine use did not substantially change (<5%) the prevalence risk of glucose impairment associated with overweight or obese BMI (**S2 Appendix**).

Diabetes awareness was low among those found to have diabetes (n = 16; 35.6%), and few individuals with diabetes were receiving biomedical treatment at the time of the initial data collection (n = 15; 33.3%). The prevalence of adults with diabetes reporting traditional medicine use was 77.1% (95% CI 58.0–89.2%).

Many individuals had end-organ micro-vascular complications associated with diabetes (**Table 2**). The prevalence of renal complications among individuals with diabetes was 12.0% (95% CI 4.7–27.3) and included reduced eGFR and albuminuria. The prevalence of ophthalmologic complications was 49.6% (95% CI 28.6–70.7) and included retinopathy and maculopathy. The prevalence of neurologic complications manifested as peripheral neuropathy was 28.8% (95% CI 8.0–65.1). The prevalence of individuals with diabetes having at least one complication was 52.3% (95% CI 34.8–69.3), and the prevalence of having multiple complications (≥2) was 33.4% (95% CI 10.7–67.8).

**Table 2:** Prevalence of end-organ micro-vascular complications present in adults with diabetes from Kilimanjaro (n = 45*; CKD-AFRiKA 2015).

Complications	N (%)	Prevalence^±^ (%; 95% CI)
**Renal**		
**Reduced eGFR^‡^**	4 (8.9%)	3.8% (1.0–14.0)
**Albuminuria**	11 (24.4%)	11.3% (4.4–26.0)
**Albuminuria or reduced eGFR**	12 (26.7%)	12.0% (4.7–27.3)
**Ophthalmological**		
**Background retinopathy**	12 (34.3%)	36.2% (14.8–64.8)
**Pre-proliferative retinopathy**	3 (8.6%)	12.3% (3.1–38.2)
**Proliferative retinopathy**	2 (5.7%)	1.1% (0.2–5.7)
**Maculopathy**	9 (25.7%)	24.6% (10.3–48.1)
**Retinopathy or Maculopathy**	17 (48.6%)	49.6% (28.6–70.7)
**Neurological**		
**Peripheral neuropathy**	7 (21.2%)	28.8% (8.0–65.1)
**≥1 complication**	25 (55.6%)	52.3% (34.8–69.3)
**≥2 complications**	9 (20.0%)	33.4% (10.7–67.8)

*Among the 45 participants with diabetes, 45 (100%) were assessed for renal complications, 35 (78%) for ophthalmological complications, and 33 (73%) for neurological complications.

±Sample-adjusted, weighted prevalence among individuals with diabetes.

‡≤60 ml/min/1.73m2 by Modification of Diet in Renal Disease formula without the race factor.

## Discussion

Among a randomly-selected, community-based sample from northern region of Tanzania, we observed a high prevalence of glucose impairment, including diabetes, which was strongly associated with obesity. Despite a burden that is comparable to several middle- and high-income countries, awareness was low, and few people were receiving biomedical treatments. Concurrently, we observed a high prevalence of diabetes-associated complications, including retinopathy, neuropathy, and nephropathy [6, 24–26].

Globally, obesity is a major risk factor for diabetes, and its prevalence is growing rapidly in LMICs [6, 27]. In SSA, rates of obesity are among the fastest growing in the world [28]. Our findings, which highlight the significant association between obesity and glucose impairment in parts of the Kilimanjaro Region of Tanzania, extend the findings from others studies across different regions of SSA showing obesity as an important, modifiable risk factor for diabetes [7, 29–34]. We also observed a high prevalence of co-morbid conditions, such as hypertension and CKD among adults with diabetes, and this further emphasizes that public health efforts addressing diabetes should incorporate shared, modifiable risk factors for several NCDs.

As in high-income countries, diabetes in SSA is a major risk factor for death and disability from cardiovascular disease, stroke, CKD, and infections, particularly soft tissue and bloodstream infections [7, 8, 10, 35]. However, despite the health consequences of diabetes in SSA, most national health systems are unprepared for the burden [3, 36]. In Tanzania, very few primary-level health centres have services directed towards NCDs such as clinical providers, diagnostics, treatments, and referral capacities [37]. Consistent with this, few participants in our study had been previously diagnosed with diabetes or were receiving biomedical treatments for diabetes, while diabetes-associated complications were common.

Notwithstanding the high prevalence of undiagnosed and untreated diabetes in parts of the Kilimanjaro Region, we observed an even higher prevalence of community members with glucose impairment who are at increased risk for developing diabetes. This finding indicates that a substantially greater disease burden may underlie what is currently known about diabetes in the region. Furthermore, considering the high prevalence of diabetes-associated complications, which are costly, resource-intensive to treat, and associated with poor health outcomes including death, this further highlights the importance of both primary and secondary prevention of diabetes through comprehensive epidemiology reporting including screening of high-risk populations, risk mitigation by reducing modifiable risk factors, preparation of health systems, and increasing public health and primary care capacity [31, 38].

Our study is among the first community-based studies assessing glucose impairment, diabetes, and diabetes-associated complications in SSA, and in Tanzania, to our knowledge, there are only two other community-based studies on diabetes in the last 15 years [29, 35, 39–43]. Moreover, our study is among the first to use HbA1C as a diagnostic test for diabetes in SSA, and we assessed for diabetes-associated complications using clinically meaningful parameters which may be more readily available in clinical or research settings across the region.

Despite these strengths, there are also limitations of our study. Given the cross-sectional design, causal relationships between diabetes and measured risk factors or common micro-vascular complications cannot be drawn. Misclassification of disease around the cut-off points for disease definitions may also be present although we expect any misclassification to be non-differential. The Bayer A1c Now+ point-of-care device that we used to measure A1c has not been validated in this population, and we defined diabetes using values based on parameters assigned from different populations. As such, it is unknown what cut-off values should be used to define diabetes and diabetes risk in this population or how these point-of-care values correspond to laboratory based measurements of HbA1c in this population. Considering the substantial and growing burden of diabetes and other NCDs in the region, validated, accessible point-of-care devices used for diagnosis and monitoring are urgently needed [7, 44].

Furthermore, we were unable to distinguish between insulin-dependent or Type I diabetes and Type II diabetes. In our study, several individuals with diabetes were normal or underweight, which may suggest that insulin-dependent or Type I diabetes is also an important health burden in the region, and further epidemiology work is needed to understand this potentially important distinction. Finally, non-response bias can be introduced when the response rate is low and when there is a substantial difference between responders and non-responders. To address non-response bias that may have arisen from differences between the respondents and non-respondents, we used sample-balanced weights for age and gender, and we explored potential differences in characteristics between the two groups. However, the additional potential for informative missingness in the retinopathy and neuropathy measurements means that our estimates for these diabetes-associated complications may be under-estimated as illness was a reported reason for non-response.

In conclusion, in parts of northern Tanzania, diabetes is an under-recognized health condition despite that fact that many people either have diabetes or are at increased risk for developing diabetes. Most individuals with diabetes or at risk for diabetes were undiagnosed or untreated, and the prevalence of diabetes-associated complications, including retinopathy, neuropathy, and nephropathy, was high. Public health efforts will need to address prevention with a focus on reducing modifiable risk factors, which appears to include obesity, as well as early detection aimed at increasing awareness. These findings highlight a growing urgency of diabetes prevention in this region as well as the need for treatment, including management of complications.

## Supporting Information

S1 Appendix Detailed Methods: Standard Operating Protocol (SOP) for Household Selection and Neuropathy Evaluation. (DOCX)

S2 Appendix Stepwise Model Specification. (DOCX)
