# Supplementary material for: Prevalence, Risk Factors, and Complications of Diabetes in the Kilimanjaro Region: A Population-Based Study from Tanzania
Source: PLoS One. 2016 Oct 6;11(10):e0164428. doi: 10.1371/journal.pone.0164428 (PMC5053499; doi:10.1371/journal.pone.0164428)
Supplement: S1 Appendix — (DOCX) [file pone.0164428.s001.docx]

**Prevalence, Risk Factors, and Complications of Diabetes in the Kilimanjaro Region: A Population-Based Study from Tanzania**

**Supplementary Appendix S1**

**Detailed Methods: Standard Operating Protocol (SOP) for Household Selection and Neuropathy Evaluation.**

John W Stanifer*, MD, MSc^1,2,3^ and Charles R Cleland*, MBBS, BSc, MD^4^; Gerald Jamberi Makuka, MD^5^; Joseph R Egger, PhD^2^; Venance Maro, MD, MMed^5^; Honest Maro, MD, MMed^4^; Francis Karia, MBA, MSc^5^; Uptal D Patel, MD^1,2,3^; Matthew J Burton, PhD^6^; Heiko Philippin MD^4,6^ for the Comprehensive Kidney Disease Assessment For Risk factors, epidemiology, Knowledge, and Attitudes (CKD AFRiKA) Study

1 Division of Nephrology, Department of Medicine, Duke University; Durham, NC United States

2 Duke Global Health Institute, Duke University; Durham, NC United States

3 Duke Clinical Research Institute, Duke University; Durham, NC United States

4 Eye Department, Kilimanjaro Christian Medical Centre, Moshi, Tanzania

5 Kilimanjaro Christian Medical University College; Moshi, Tanzania

6 International Centre for Eye Health, London School of Hygiene & Tropical Medicine, London, United Kingdom

*Co-first authors; each author contributed equally to this manuscript

Correspondence:

John W Stanifer, MD, MSc

Duke University Medical Center

Box 3182

Durham, NC United States 27710

Email: john.stanifer@duke.edu

Phone: 423 526 7113

Fax: 919 681 6448

**Household Selection**

PURPOSE:

To provide a reproducible, systematic, and random method of selecting households for sampling.

DEFINITIONS:

- Cluster = randomly, pre-selected geographic location that includes multiple households for sampling
- Dwelling= A free-standing building that is covered by a roof. Buildings that share a foundation or appear to share a foundation should be considered as one dwelling.
- Household = Persons residing within a dwelling whose food is prepared by the same person(s)
- ID = Unique Identification Number that is assigned to each participant and each household.
- Household ID= Two digit Unique Identification Number contained in the study ID number that is assigned to each household.
- Eligible Individuals: Adults over the age of 18 who are not pregnant. Ex-pats or Temporary Residents should be excluded unless they are FULL citizens who reside in Tanzania full time (i.e. more than 9 months out of every year).

OVERVIEW:

- Cluster site identification
- Household identification
- Household selection process

PROCESS:

1. Cluster site identification: the starting point from which household selection will occur has been identified based on a random GPS coordinates.
2. The dwelling physically closest to the starting point will be approached first.
3. Household Selection Process
   1. The first dwelling should be approached:
      1. If that dwelling fulfills the definition of a household then assign it a household ID and assess the eligibility of the household adults according to the enrollment protocol.
      2. If that dwelling does NOT fulfill the definition of a household then move on to the next dwelling.
      3. Unless the dwelling is clearly marked as a business, shop, or restaurant then the field surveyors should assume that it could be a household. They should then approach to confirm. (*Remember that sometimes people who own shops also live in the back – if any doubt then they should always approach to confirm*).
   2. To identify the next dwelling to approach for sampling, the following methods should be used:
      1. The field surveyor will stand with his/her back to the main entrance of the first dwelling.
      2. Flip a coin.
      3. If the coin lands on TAILS then proceed to your LEFT. If the coin lands on HEADS then proceed to on your RIGHT.
      4. Next, roll the die to determine which house to approach. The numbers on the die represent which house number (in sequential order according to physical distance to the front door) will be chosen.
      5. If the surveyor comes to an intersection or dead-end before reaching the house number on the die, then flip the coin again to determine the continuing direction. Again, TAILS will be LEFT and HEADS will be RIGHT.
      6. In instances where there is only one physical direction to go, then proceed in that direction.
      7. If a dwelling repeats, then repeat the coin-flip and die process.
4. Protocol for Gated Houses
   - - - 1. House with a Gatekeeper

First, contact the gatekeeper to explain our intentions. If agreeable, he may allow entry.

If not agreeable to entry, then leave a study overview pamphlet along with our contact information.

Arrange a follow-up time to see if the owners have expressed interest.

- - - - 1. Closed Gate House

If a gatekeeper is present then proceed as above.

If no gatekeeper and no way to contact the household members, then record as non-response.

Two additional visits, including one off-hours visit (i.e. evening or weekend day), should be attempted according to the follow-up protocol.

- - - - 1. Open Gate

First, ensure that there is no gatekeeper.

If no gatekeeper, then approach the household as you would any other dwelling.

**Neuropathy Evaluation**

PURPOSE:

To provide a reproducible, systematic method for evaluating distal lower extremity peripheral neuropathy.

OVERVIEW:

- Examiner
- Equipment/Supplies
- Assessment
- Grading

PROCESS:

1. The same physician will examine all participants for distal, lower-extremity peripheral neuropathy using a scored combination of vibration perception, pinprick sensation, and Achilles ankle reflexes
2. Equipment:
   1. 128-Hertz tuning fork
   2. 10-g monofilament
   3. Reflex Hammer
3. **Vibration perception assessment**
   1. First, after striking the tuning fork, place it on the participant’s forehead to establish the baseline (normal) sensation.
   2. Second, after striking the tuning fork place the tuning fork, place it on the dorsum of the inter-phalangeal joint of the right hallux.
   3. Ask the participant to state when he/she can no longer perceive the vibration sensation.
   4. Record the participant’s response as either normal sensation (0), diminished sensation (0.5), or absence of sensation (1).
      1. Normal sensation: participant correctly identifies the cessation of the vibration sensation
      2. Diminished sensation: participant incorrectly or is delayed in his/her perception of the cessation of vibration sensation
      3. Absence of sensation: participant is unable to perceive vibration sensation
   5. Repeat the process on the dorsum of the right hallux.
4. **Pinprick sensation**
   1. The assessment should be done in a quite setting and the participant should not be able to see where the examiner applies pressure.
   2. First, using the monofilament, apply pressure to the participant’s forehead to establish the baseline (normal) sensation.
      1. Apply sufficient pressure to cause the monofilament to bend and the skin to indent.
      2. The total duration of the skin contact should not exceed 2 seconds.
   3. Second, using the monofilament apply pressure to the distal plantar aspect of both halluces and each metatarsal joint.
      1. Apply sufficient pressure to cause the monofilament to bend and the skin to indent.
      2. The total duration of the skin contact should not exceed 2 seconds.
      3. Do not allow the monofilament to slide across the skin or make repetitive contact at the same site.
   4. Record the participant’s response at each site as either normal sensation (0), diminished sensation (0.5), or absence of sensation (1).
5. **Achilles ankle reflexes**
   1. First, the participant should be seated erect at 90 degrees, in a relaxed position, with his/her legs hanging off the edge of the examination table/seat. The participant’s feet should not be touching the floor, and the participant’s calf muscles should be visible.
      1. If the participant cannot maintain this position, then have him/her lie supine crossing one leg over the other in a figure four position.
   2. Second, identify the Achilles tendon. Confirmation of the tendon location can be achieved by having the participant perform plantar flexion.
   3. Third, support the foot so that it forms a right angle with the rest of the lower leg. With the Achilles tendon stretched and the ankle in a neutral position, strike the tendon with the reflex hammer.
      1. A normal reflex should cause the calf muscle to contract, and the foot to plantar flex.
   4. If the initial response was negative, the patient was examined again with reinforcement.
   5. Record the participant’s response for each Achilles tendon as either normal sensation (0) or absence of sensation (1).
6. Based on these three assessments, a total score (0-8) will summed for each participant.
   1. A total score was ≥4 will be considered positive for peripheral neuropathy.
