# Supplementary material for: Prevalence, Risk Factors, and Complications of Diabetes in the Kilimanjaro Region: A Population-Based Study from Tanzania
Source: PLoS One. 2016 Oct 6;11(10):e0164428. doi: 10.1371/journal.pone.0164428 (PMC5053499; doi:10.1371/journal.pone.0164428)
Supplement: S2 Appendix — (DOCX) [file pone.0164428.s002.docx]

**Prevalence, Risk Factors, and Complications of Diabetes in the Kilimanjaro Region: A Population-Based Study from Tanzania**

**Supplementary Appendix S2**

**Detailed Methods: Step-wise model specification**

John W Stanifer*, MD, MSc^1,2,3^ and Charles R Cleland*, MBBS, BSc, MD^4^; Gerald Jamberi Makuka, MD^5^; Joseph R Egger, PhD^2^; Venance Maro, MD, MMed^5^; Honest Maro, MD, MMed^4^; Francis Karia, MBA, MSc^5^; Uptal D Patel, MD^1,2,3^; Matthew J Burton, PhD^6^; Heiko Philippin MD^4,6^ for the Comprehensive Kidney Disease Assessment For Risk factors, epidemiology, Knowledge, and Attitudes (CKD AFRiKA) Study

1 Division of Nephrology, Department of Medicine, Duke University; Durham, NC United States

2 Duke Global Health Institute, Duke University; Durham, NC United States

3 Duke Clinical Research Institute, Duke University; Durham, NC United States

4 Eye Department, Kilimanjaro Christian Medical Centre, Moshi, Tanzania

5 Kilimanjaro Christian Medical University College; Moshi, Tanzania

6 International Centre for Eye Health, London School of Hygiene & Tropical Medicine, London, United Kingdom

*Co-first authors; each author contributed equally to this manuscript

Correspondence:

John W Stanifer, MD, MSc

Duke University Medical Center

Box 3182

Durham, NC United States 27710

Email: john.stanifer@duke.edu

Phone: 423 526 7113

Fax: 919 681 6448

| Table 3. Stepwise model specification. Model-predicted prevalence risk ratios (PRR) for glucose impairment associated with overweight/obese status (CKD-AFRiKA 2015). | | | | | | |
| --- | --- | --- | --- | --- | --- | --- |
| Model (co-variate) | **Model Parameters** | | | | | |
|  | Beta coefficient | (Bias)^2^ | Standard error | Variance | Mean square error | PRR (95% CI) |
| 1 (overweight/obese) | 0.651 | 0.00 | 0.268 | 0.0718 | 0.0718 | 2.16 (1.39-3.36) |
| 2 (overweight/obese + gender) | 0.759 | 1.17E-02 | 0.245 | 0.0600 | 0.0717 | 2.14 (1.13-3.51) |
| 3 (overweight/obese + gender + ethnicity) | 0.790 | 1.93E-02 | 0.271 | 0.0734 | 0.0928 | 2.20 (1.27-3.82) |
| 4 (overweight/obese + gender + ethnicity + age) | 0.726 | 5.62E-03 | 0.424 | 0.1798 | 0.1854 | 2.06 (1.36-3.14) |
|  |  |  |  |  |  |  |
